# The Prevention of Tuberculosis in Ireland

**Published:** 1908-10-31

**Authors:** 


### THE PREVENTION OF TUBERCULOSIS IN IRELAND.

The Bill brought in by Mr. Birrell to prevent the spread, and provide for the treatment of, Tuberculosis in Ireland came before Standing Committee A of the House of Commons on October 21. The Bill provides in the first clause for compulsory notification by the medical practitioner to the medical officer of health of the district in the case of any person suffering from tuberculosis of any form or at any stage. A number of amendments were proposed and discussed to limit the scope of the provision. Lord Balcarres suggested that the only satisfactory way of dealing with the matter was to institute fines for public spitting. Mr. Birrell replied that there was a strong feeling against spitting in Ireland, and he quite agreed that it ought to be put down. Mr. Seaverns remarked that penalties against spitting had been productive of admirable results in Sydney and New York. An amendment, proposed by Mr. C. C. Craig, was that notification should be left to the discretion of the medical practitioner in particular cases. Mr. Birrell opposed this, adding that the Irish Local Government Board would in its regulations be animated by a desire to deal only with cases in an advanced stage; and Mr. T. W. Russell subsequently stated that the principal object of the Board would be to make tuberculosis compulsorily notifiable only in such cases as could be helped. Only cases of open tuberculosis of the lung would at first be notifiable, and then only when the patient was living under conditions dangerous to other people. The amendment was rejected by thirteen to nine. The Bill was still under consideration when the Committee adjourned.

